# Supplementary material for: Identification of photoperiod-induced specific miRNAs in the adrenal glands of Sunite sheep (Ovis aries)
Source: Front Vet Sci. 2022 Jul 22;9:888207. doi: 10.3389/fvets.2022.888207 (PMC9354845; doi:10.3389/fvets.2022.888207)
Supplement: Supplementary file 1 [file Data_Sheet_1.ZIP › Supplementary file/miRDeep2 file.zip!/miRDeep2 file/Novel_Structure/LP42A2/Novel_112.pdf]

Provisional ID	:	NC_019482.2_35972
Score total	:	1.7
Score for star read(s)	:	-1.3
Score for read counts	:	0
Score for mfe	:	1.4
Score for randfold	:	1.6
Score for cons. seed	:	
Total read count	:	173
Mature read count	:	173
Loop read count	:	0
Star read count	:	0

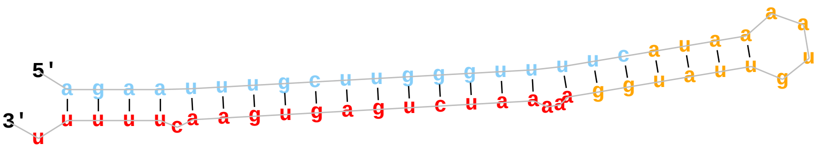

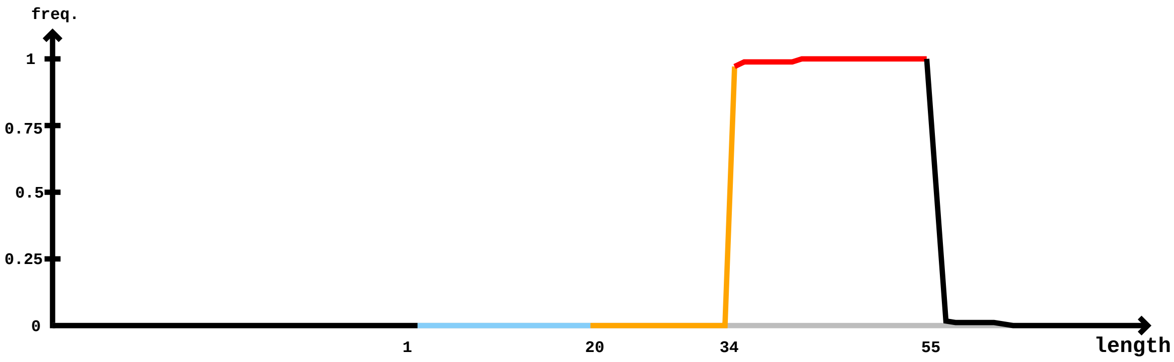


```
                                                    Star                       Mature
5'- aaccauuauauacagaugauuuagugaguuggccaaagaauuugcuuggguuuucauaaaauguuauggaaaaaucugagugaacuuuuuggccaacggaauauuuuucucc   -3'    exp
    ...(((((.............))))).((((((((((((.(((((((((((((((((((....)))))))..)))))))))))).))))))))))))((((.....))))..    reads    mm    sample
    .....................................................................aaaaaucugaAugaacuuuuu......................    111      1     seq
    .....................................................................aaaaaucugaAugaacuuuuug.....................    59       1     seq
    ......................................................................aaaaucugagugaacuuuuu......................    1        0     seq
    ......................................................................aaaaCcugagugaacuuuuug.....................    1        1     seq
    ......................................................................aaaaucugagugaacuuuuugg....................    1        0     seq
    ............................................................................ugagugaacuuuuuggccaaG...............    1        1     seq
    ............................................................................ugagugaacuuuuuggccaacU..............    1        1     seq
```
